# Supplementary material for: Persistence of sleep difficulties for over 16 years amongst 66,948 working-aged adults
Source: PLoS One. 2021 Nov 18;16(11):e0259500. doi: 10.1371/journal.pone.0259500 (PMC8601511; doi:10.1371/journal.pone.0259500)
Supplement: S1 File — (DOCX) [file pone.0259500.s001.docx]

Supplement 1. Description of the dataset

| Variable | Description |
| --- | --- |
| id | Research id |
| year | Year of wave |
| type | 1=still employed by the organization; 2=left from the organization |
| sex | 1=man; 2=woman |
| age | Age at the time of wave |
| sleephrs | Sleep hours per day: 1=<6h; 2=6.5h; 3=7h; 4=7.5h; 5=8h; 6=8.5h, 7=9h; 8=9.5h; 9>=10h |
| jenk1 | Jenkins Sleep Scale Item #1 |
| jenk2 | Jenkins Sleep Scale Item #2 |
| jenk3 | Jenkins Sleep Scale Item #3 |
| jenk4 | Jenkins Sleep Scale Item #4 |
| bmi | Body Mass Index |
| smoke | Current smoking: 0=no; 1=yes |
| alcog | Weekly alcohol consumptions in grams |
| met | Weekly physical activity in MET-h |
